# Evaluation of the advanced micro-scale bioreactor (ambr™) as a highthroughput tool for cell culture process development

**DOI:** 10.1186/1753-6561-7-S6-P73

**Published:** 2013-12-04

**Authors:** Frédéric Delouvroy, Guillaume Le Reverend, Boris Fessler, Gregory Mathy, Mareike Harmsen, Nadine Kochanowski, Laetitia Malphettes

**Affiliations:** 1 Cell Culture Process Sciences, Biotech Sciences, UCB Pharma S.A., Chemin du Foriest, Braine l'Alleud, Belgium

## Introduction

Bio-pharmaceutical industries face an increasing demand to accelerate process development and reduce costs. This challenge requires high throughput tools to replace the traditional combination of shake flasks and small-scale stirred tank bioreactors. A conventional and widely used process development tool is the stirred tank reactor (STR) ranging from approximately 1L to 10L in working volume. Physical culture parameters such as pH, temperature and pO_2 _can be easily controlled in such systems.

However preparation and operation of these systems are time and resource consuming. The ambr™ system from TAP Biosystems has the capabilities for automated sampling, feed addition, and control for pH, dissolved oxygen, gassing, agitation, and temperature.

Here, through the evaluation of parameters including cell growth, viability, metabolite concentration and production titer during a fed-batch process using CHO cells producing a recombinant mAb, we assessed the reproducibility of the ambr™ system for standard conditions compared to 2L stirred tank bioreactors and the effects of parameter ranging between both culture systems, namely feed rate and pH ranging.

## Material and methods

A CHO cell line expressing a recombinant monoclonal antibody was used. Cells were carried out for 14 days in a fed-batch mode in a chemically defined medium and fed according to process description.

Culture systems: ambr™48 is an automated system with 48 disposable microbioreactor vessels. Results of ambr™ 48 workstation (TAP Biosystems) were compared to the results obtained with 2L stirred tank bioreactors with Biostat B-DCUII control systems (Sartorius Stedim).

Commercially available production media and feeds were used as per manufacturer's recommendations. pH (7.0 +/- 0.2 for standard conditions). All fed-batch cultures lasted 14 days.

For the scale down model, parameters were divided in two groups. 1. The scale dependent factors: culture start volume, feed volumes that are linearly dependent and agitation speed and gazing that are theoretically or by experiences determined. 2. The scale independent factors: Media, temperature, seeding densities, pH, dissolved O_2_, culture duration.

Product quality of the monoclonal antibody produced was analyzed as follows: Cell culture fluid samples were centrifuged and filtered to remove cell debris. The monoclonal antibody was purified by ÄKTA-express (GE Healthcare) Protein-A purification. The neutralized eluate was used for product quality analysis.

Sample analysis: Viable Cell Concentration (VCC) and cell viability were measured using a ViCell XR cell counter (Beckman Coulter). Metabolite concentrations were measured by enzymatic assay using a UV-method (R-Biopharm) for the ambr™ vessels and by a BioProfile Analyzer 400 (Nova Biomedical) for stirred tank bioreactors. For both systems, pH measurement was obtained with a BioProfile pHOx pH/Gas Analyzer (Nova Biomedical), Osmolality was obtained using a Omometer (Advanced Instruments). Production titers were measured throughout the culture using an Octet QK (ForteBio) and after 14 days with protein A HPLC (Agilent) after purification.

Design of experiment: A 3x7-factorial design was implemented using JMP software (SAS). Parameter ranging included pH (6.9, 7.0, and 7.1) and feed rate addition (±30%, ±20% and ±10% compared to standard conditions) see Table 1

**Table 1:** Design of the experiment

pH set point	Feed rate	**Number of replicates in ambr**™ **run**	Number of replicates in 2L bioreactor run
7.0	-30%	2	0

7.0	-20%	2	1

7.0	-10%	2	1

7.0	Control feed rate	6	1

7.0	+10%	2	1

7.0	+20%	2	1

7	+30%	2	0

6.9	Control feed rate	2	1

7.1	Control feed rate	2	1

## Results and discussion

The ambr™ run was performed in parallel to a 2L bioreactor run. Both experiments were inoculated with the same pool of cells, same batches of media and feeds were used in both systems. Different pH setpoints and feed rates were assessed to determine the impact on cell growth (see Table 1), viability and mAb titers. Each condition was tested in duplicates in the ambr ™ minibioreactors and singlet in 2L bioreactors. The design of experiment is described in Table 1. The aim of this experiment was to test the reproducibility within ambr™ and the comparability between the minibioreactors and the 2L.

**Figure 1:**
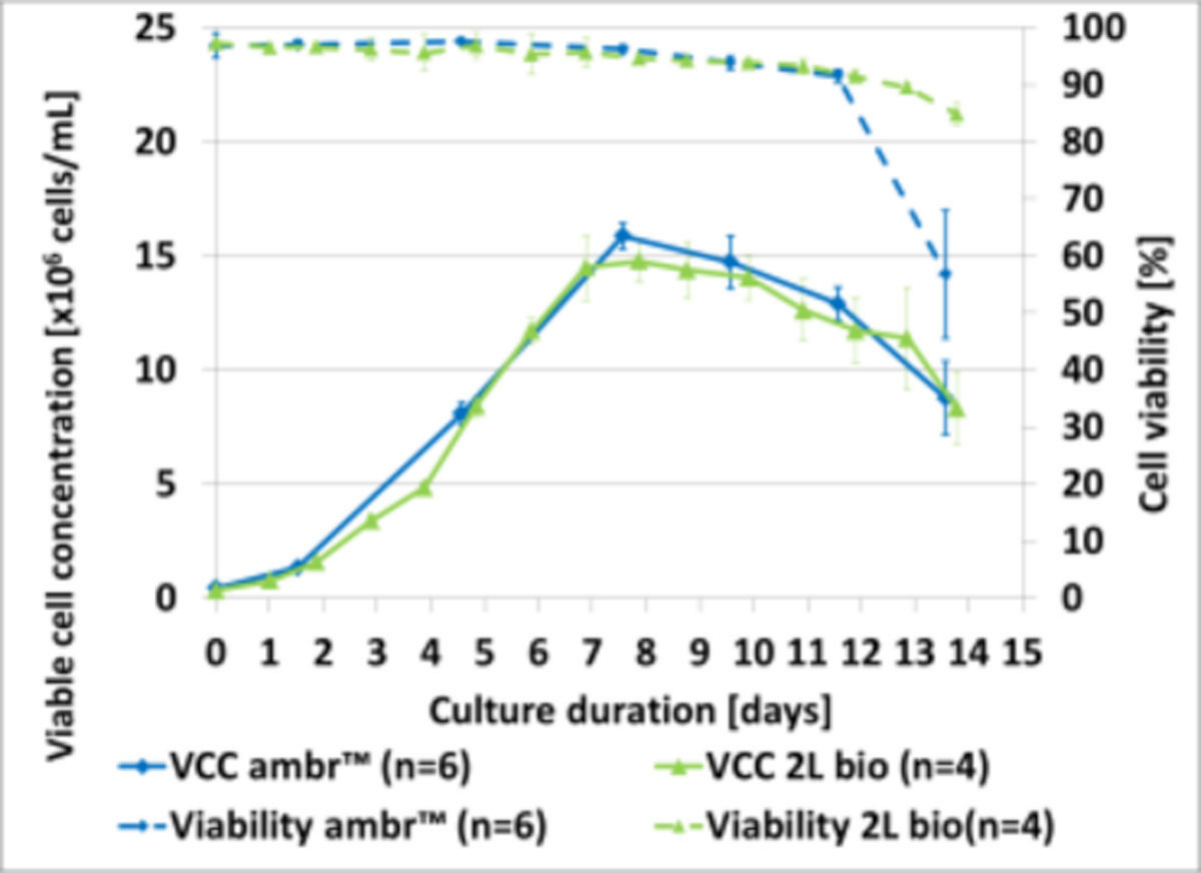
Viable cell concentration (VCC) and viability average comparison between ambr ™ and 2L bioreactors (control runs)

Cell growth and cell viability were monitored daily throughout the cultures in 2L (control runs, n = 4). In the ambr™ system, cell density and viability were measured every two days to avoid excessive sampling on control runs (n = 6). Cell viabilities were maintained at acceptable values (>80%) throughout the cultures in the established culture conditions.(Figure 1). Cell growth and viability performances observed in the ambr™ minibioreactors and 2L bioreactors were comparable (Figure 1). Final mAb titer obtained using ambr™ showed slightly (15%) lower concentration than the 2L bioreactors. Osmolality profiles showed the same trend in 15mL and 2L bioreactors (between and 300 mOsm/kg at the beginning and 420mOsm/kg at the end of the run). Online pH profiles were also comparable in both ambr™ minibioreactors and in 2L bioreactors.

The impact of different feed rates were assessed and compared between 2L bioreactors stirred tank bioreactors and ambr™ minibioreactors. Obtained results show similar profiles of viable cell density, cell viability, pre-harvest Mab titer at day 14 and osmolality profiles with different feed rates.

High feed rates and low feed rates impact cell growth profiles and osmolality profiles. The different feed rates applied do not show any significant impact on the final mAb titer. Profiles observed in 2L bioreactors and ambr™ are comparable in both systems, except viability at the end of the ambr™ run due to a lack of glucose.

The impact of different pH setpoints on cell growth, viability, final mAb titer and osmolality didn't showed significant impact on those parameters in both systems. mAb titer at day 14 was comparable in 2L stirred bioreactors than in the ambr™ system.

## Conclusions

Our evaluation of the ambr™ system showed there is good reproducibility within the 6 ambr™ controls. There is good comparability in terms of cell growth, product titer, pH, pO2 and osmolality profiles as well as PQA obtained between ambr™ and bioreactors despite the fact ambr™ used a bolus feeding regimen and the stirred tank bioreactors used a continuous feeding strategy. The impact of feed rate on cell growth and osmolality upon feed rate ranging was observed in both culturing systems, but has no impact on PQA. pH set point ranging did not have an impact on the measured output parameters in either scale. ambr™ provides a predictive and resource-efficient tool to do process development especially media testing, feeding strategy screening and cell culture production conditions.

